# New Internet of Medical Things for Home-Based Treatment of Anorectal Disorders

**DOI:** 10.3390/s22020625

**Published:** 2022-01-14

**Authors:** Jerry Zhou, Vincent Ho, Bahman Javadi

**Affiliations:** 1 Translational Gastroenterology Laboratory, School of Medicine, Western Sydney University, Campbelltown, NSW 2560, Australia; j.zhou@westernsydney.edu.au (J.Z.); v.ho@westernsydney.edu.au (V.H.); 2 Department of Gastroenterology, Campbelltown Hospital, Campbelltown, NSW 2560, Australia; 3 School of Computer, Data and Mathematical Sciences, Western Sydney University, Penrith, NSW 2751, Australia

**Keywords:** home healthcare, rehabilitation, biofeedback therapy, Internet of Medical Things (IoMT), medical sensors

## Abstract

Home-based healthcare provides a viable and cost-effective method of delivery for resource- and labour-intensive therapies, such as rehabilitation therapies, including anorectal biofeedback. However, existing systems for home anorectal biofeedback are not able to monitor patient compliance or assess the quality of exercises performed, and as a result have yet to see wide spread clinical adoption. In this paper, we propose a new Internet of Medical Things (IoMT) system to provide home-based biofeedback therapy, facilitating remote monitoring by the physician. We discuss our user-centric design process and the proposed architecture, including a new sensing probe, mobile app, and cloud-based web application. A case study involving biofeedback training exercises was performed. Data from the IoMT was compared against the clinical standard, high-definition anorectal manometry. We demonstrated the feasibility of our proposed IoMT in providing anorectal pressure profiles equivalent to clinical manometry and its application for home-based anorectal biofeedback therapy.

## 1. Introduction

Home healthcare has become the fastest growing sector in the healthcare industry [1,2]. Its global market is valued at USD 303.6 billion in 2020, having grown at a compound annual rate of 8.1% since 2014 [3]. Home healthcare coincided with the emerging trend in patient-centred care, coined by the Picker/Commonwealth Program in 1988. This model focuses on patients’ individual healthcare needs though active patient participation and partnership with health providers [4]. Home healthcare provides a simple extension of the clinical sessions to enable patients to be an active participant in their own treatment, this is particularly attractive for low-risk but resource-intensive rehabilitation therapies. The rapid adoption of telemedicine over the COVID-19 global pandemic has also seen a surge in consumer demand for technologies to support treatment from home. However, despite its benefits, home healthcare has seen slow adoption into clinical practice due to provider concerns over patient compliance and optimal performance of therapies at home. In order to truly integrate clinics with patients in patient-centred care, there is an opportunity to leverage emerging technologies such, as Internet of Things (IoT) [5], to address these barriers for adoption. IoT offers a seamless platform where digital devices can make sense of information without human intervention. There were an estimated 24 billion interconnected devices in 2020; the main advantage of such a huge number of connected devices is access to big datasets, which can be utilized in smart applications, such as precision healthcare [6]. The application of IoT in the healthcare domain is called Internet of Medical Things (IoMT), which provides significant benefits for health and wellbeing of people by increasing their quality of life and reducing healthcare costs [7].

Anorectal biofeedback (BF) is a first-line rehabilitation therapy for the treatment of anorectal muscle disorders [8,9]. These disorders cause 33% of chronic constipation and 60% of faecal incontinence [10,11], 2 of the most prevalent gastrointestinal disorders, affecting 16% of the global population [12,13]. BF therapy uses instrument-based “operant conditioning” (learning through repetition and reinforcement) to restore the normal muscle patterns for defecation. BF therapy is recommended by several consensus groups, including the American and European Neurogastroenterology and Motility Societies [9]. Randomized clinical trials demonstrate BF efficacy of 75–89% in long-term symptom improvements [14,15] and 83% reported improvements to quality of life [16]. Effective BF therapy demands regular attendance to a clinic over the treatment period of 6–8 weeks. Each session involves inserting a probe into the patient’s anal canal, which is then connected to a display for real-time visual feedback of muscle activities. Despite its benefits, BF is limited to few specialized clinics in tertiary hospital, due to the following challenges:

(1) Rising medical costs: The high cost of equipment and technical staff required to deliver BF makes it difficult to establish clinics in regional and community hospitals.
(2) Time consuming: Average BF therapy involves the 6 weekly sessions and follow-up sessions after 6 and 12 months. Despite demand, clinics are limited by the number of patients they can treatment at one time.
(3) Adherence: Difficulties accessing BF and the relatively invasive nature of the procedure means fewer than 30% of patients are able to adhere and complete their prescribed treatment.

Home-based BF with training devices has been proposed in past studies [17,18,19]. These have demonstrated equivalent efficacy to clinic-based therapy, while being more cost effective. However, due to technical limitations, these past devices have not had the capabilities for providers to remotely monitor and track patient compliance, which has significantly limited their adoption into clinical practice.

In this paper, we propose a new IoMT to deliver home-based BF for patients with anorectal disorders. Our system consists of an insertable probe with two force sensors to collect data on anorectal muscle contractility and coordination. The probe is wirelessly connected to a mobile app to provide real-time visual feedback of muscle activities to the user. The app records and processes training session data and clinical indicators (symptoms and bowel diary) from user input. Processed data will be sent to a cloud server to allow health providers to track progress and aid in decision making. From our experimental results, the proposed IoMT is able to record BF parameters and pressure profiles, comparable to the clinical standard—high-definition anorectal manometry (HD-ARM). It was also able to provide reproducible data over consecutive days of training. The main contributions of this paper are as follows:

- Designing a new IoMT for home-based treatment of anorectal disorders using a user-centric approach.
- Development and implementation of hardware and software components of the proposed IoMT including a new sensor in a modular architecture.
- Evaluation of the proposed IoMT in terms of accuracy and consistency in a real working condition.

The rest of this paper is organized as follows. Related work is described in Section 2. In Section 3, we present the system analysis and design, including the proposed system model and its components. Section 4 includes the detail of the proposed system architecture and its implementation. The performance evaluation and results are presented in Section 5. Conclusion and future work are presented in Section 6.

## 2. Related Work

Internet of Medical Things (IoMT) comprises of interconnected devices and applications in medical and healthcare information technology in order to improve patients’ health and wellbeing [20]. With the recent advancements in the field of artificial intelligence (AI), telemedicine, and sensor technology, IoMT can be used for wide range of medical applications and clinical decision making. There are several IoMT applications, including wearable and non-wearable sensors, which have been implemented for smart patient monitoring [7]. These solutions are using new and innovative IoMT technologies to collect physiological data by directly interacting with the human body. In this work, we consider the application of IoMT for anorectal BF to improve the quality of care and reduce the associated healthcare costs.

An anorectal BF device requires four essential components; a probe, a pressure recording unit, a unit to display the recording, and a data storage facility. A range of sensor types are utilized to measure and visualize anorectal muscle activity, the most common being pressure-based manometry, anal electromyogram (EMG), defecation training balloon [9], and anorectal ultrasound [21]. High-resolution anorectal manometry (HR-ARM) and HD-ARM probes are the most commonly used systems for clinic-based anorectal diagnostics and BF therapy [22,23]. These catheters have 16–36 circumferential pressure sensors that straddle the entire anal canal and a proximal sensor placed in the rectum. These sensors provide continuous and dynamic spatiotemporal mapping of anorectal pressures. During anorectal BF therapy, HR-ARM and HD-ARM can be used to provide visual feedback to the patient and record exercise parameters to establish baseline or evaluate progress [24]. The key BF manoeuvres are the following:

(1) Rest: After probe placement, a period of time is given for the patient to relax, so that the anal sphincter tone returns to basal levels. Anal resting pressure is measured using the averaged resting pressure over the length of the anal canal over a period of 20 s. This pressure is a composition of the internal and external anal sphincter and, to a lesser extent, the hemorrhoidal plexus.
(2) Squeeze and hold: The patient is asked to squeeze the anus for as long as possible, for a maximum of 30 s. By convention, this manoeuvre is performed three times. Both the maximal contractile pressure and endurance times are measured. Ideally, rectal pressure should not increase, because that would imply the patient has contracted the abdominal wall.
(3) Simulated defecation (push): The patient bear down as if to defecate and attempt to expel the catheter. This manoeuvre should produce sufficient rectal propulsion pressures with simultaneous anal sphincter relaxation (>20% relaxation).

Although HR-ARM and HD-ARM have yielded important contributions to the understanding of anorectal physiology, they are not well suited for BF therapy due to the high setup and ongoing costs (~$75,000 USD per system; $300 USD in consumables per use). In addition, BF therapy does not require the high-fidelity data provided by these systems, which can be counterproductive in helping the patient to understand their training. Home-based BF has been proposed to be a more cost effective and accessible approach to deliver BF therapy. Several home BF devices have been developed, utilizing pressure sensors or EMG.

### 2.1. Pressure Sensors

There are two types of pressure-based probes currently used by home BF devices: (i) a solid-state probe with strain gauge transducers, and (ii) water or air pressure probes. Both probes typically have 1 or 2 sensors, evenly spaced 10–30 mm from the anal verge, and a probe diameter of 5–15 mm. Pressure-based probes with sensors located in both the anal canal and rectum are ideally suited for BF therapy; this has the advantage of displaying the anal sphincter and rectal pressure changes simultaneously, thus enabling simulated defecation training, which requires coordinated generation of rectal propulsive forces with anal sphincter relaxation.

### 2.2. Electromyography

An EMG biofeedback system consists of a surface EMG electrode that is mounted on a probe or affixed to the surface of the external anal sphincter muscle. These electrodes pick up EMG signals from the surface of the anal sphincter muscle and provide both visual feedback on the monitor and auditory pitch signals, corresponding to changes in the electrical activity of the anal sphincter. Unlike pressure sensors, EMG systems do not provide information on rectal propulsive forces, but are cheaper, more durable, and provide information on striated anal muscle activity. A meta-analysis found studies using pressure-based BF produced superior outcomes compared with studies using EMG (Chi-squared = 5.60; *n* = 717; *p* = 0.02) [25]. Overall, the success rate of BF for defecation disorders was 69–78%, regardless of the instrumentation used. The main predictor of successful outcomes for BF therapy was patient compliance [26,27].

A summary of home anorectal BF devices, either commercially available or involved in clinical studies, is presented in Table 1. The physiological information provided by home BF systems are several orders of magnitude less than that of HR-ARM and HD-ARM. However, this is not a limitation for anorectal BF therapy, where, unlike anorectal diagnostics, a high level of fidelity is not essential. Home BF systems have reported promising efficacy studies, comparable to clinical BF [17,18,19,28]. The common shortcoming of these systems is that the assessment of patient compliance to exercise programs is based solely on verbal reports or home exercise diaries [29,30]. Even systems that retain data require the user to attend a clinical appointment for information to be download and reviewed by their health provider [19]. As mentioned earlier, these uncertainties around patient compliance and quality of exercise mean that existing devices have not been widely adopted into clinical practice. In this paper, we address the limitations in the current home BF systems by proposing a new IoMT, based on force sensors and a mobile app with the ability for clinicians to monitor patient progress.

## 3. System Analysis and Design

In order to develop system and user requirements, we employed a user-centric approach by utilizing participatory design and co-design strategies [34]. In this stage, the target users were determined and the user needs were specified. A group of 38 anorectal BF patients (aged between 25 and 74 years old) from anorectal clinics were interviewed. An in-depth interview was arranged in terms of (i) basic health information, (ii) BF experience and challenges, (iii) internet and mobile phone use, and (iv) opinions on home BF devices and potential challenges. The interviews showed that patients were generally positive towards home-based therapy (87% were likely or very likely to try), and the elderly had adequate knowledge on how to use digital technology. A summary of user pain points and needs, identified through our interviews, are presented in Table 2.

Similar interviews were conducted with providers that included 14 clinical staff (8 gastroenterologists and colorectal surgeons specialized in anorectal motility disorders, and 6 specialist nurses that perform clinical BF) from 6 anorectal clinics across Sydney, Australia (Camden, Strathfield Private, St. George, Royal Prince Alfred, Royal North Shore, and Liverpool Hospitals). An in-depth interview was arranged in terms of (i) BF experience and challenges, (ii) considering of home therapy, (iii) experience with home BF devices, and (iv) opinion of existing home BF devices and barriers for their adoption into clinical practice. The interviews showed that most health providers have considered home therapy (86%), but no clinic had adopted it into clinical practices due to limitations of current home BF devices. The summary of provider pain points and needs, identified through our interviews, are summarized in Table 2.

Based on the qualitative user research, we proposed a home BF system to align with the needs and requirement of users and providers. We used the reference IoMT ecosystem, which includes three main components: data acquisition, IoMT gateway, and cloud server [7]. Data acquisition is where the data is collected from patients via sensors. The gateway can be a physical device or software program to connect sensors to the cloud and data system, so it works as a communication bridge in the system. The data will be stored and processed in the cloud server and then used by medical experts. Having a gateway will provide more flexibility to collect and pre-process the data and enable patients to protect their privacy.

**Figure 1:**
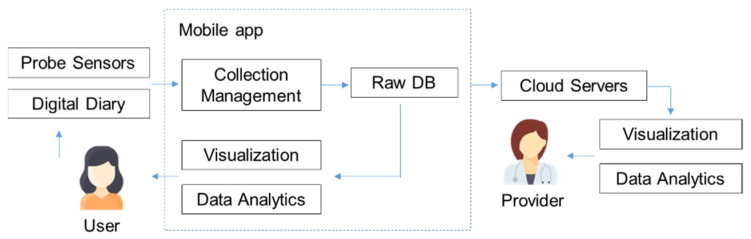
The components of the proposed IoMT.

The components of the proposed IoMT are presented in Figure 1. The data acquisition is composed of an insertable probe, where sensors capture rectal and anal sphincter pressures. Users also have the option to enter diet, symptom, and stool data into a digital diary (this would replace the physical diary required for clinical BF). To improve upon the usability of previous devices, our probe is wirelessly connected to the users’ mobile phone via a dedicated app, which is the IoMT gateway. Data is collected in the app and can be sent to cloud servers (with user permission), where it is stored and can be accessed by nominated health providers using a web application. The collected data will be used for patient treatment and to create a dataset for future research activities.

The intended use of the proposed IoMT system is to allow for daily BF sessions (5–15 min). The inserted probe provides real-time data to the app to allow for visual feedback of the user’s muscle activities. This information is used to guide the user during therapy to achieve their clinical goals (e.g., muscle strengthening).

## 4. System Architecture and Implementation

In this section, we will explain the proposed architecture for the new IoMT and the detail of development and implementation.

### 4.1. System Architecture

**Figure 2:**
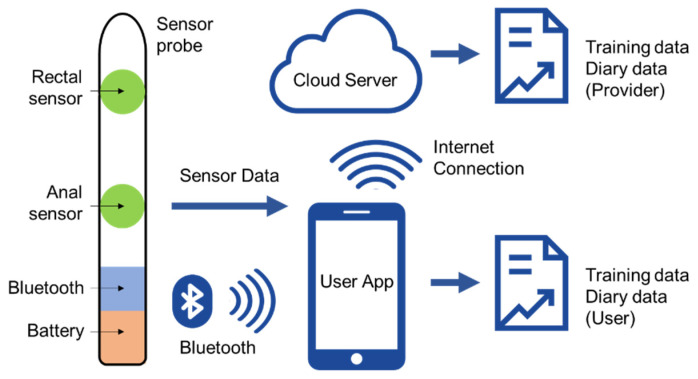
The proposed system architecture for the new IoMT.

Our proposed IoMT can be divided into four layers, as depicted in Figure 2: sensing, networking, service, and application.

(1) Sensing layer: Integrates a new sensor array, inserted intra-anally into the patient to collect data from the anorectal muscle activity. The sensors should be small enough to fit within a probe and allow for the measurement of changes in intra-luminal pressures in the rectum and the force applied by the anal sphincter muscles.
(2) Networking layer: Offers networking support and data transfer in the wireless network. We utilized short-range wireless technology (Bluetooth) to transmit our sensor data to a patient’s smart phone app.
(3) Service layer: Creates and manages all types of services, aiming to satisfy user requirements. The data collection and processing are tailored to the requirement of the patient’s therapy, such as ensuring correct exercises are performed, adjusting training intensity to be consistent with ability, and comparing current performance with baseline data. Processed data are also synced to the cloud server, allowing data analytics to discover new knowledge to improve the patient care.
(4) Application layer: Provides an easy interface that allows users to retrieve the outputs and to understand the meaning of the outputs. Simple training is provided for understanding graphics and patients can quickly check their progress. Output can also be accessed remotely by their health provider through the cloud-based web application to monitor progress and to aid in decision making.

### 4.2. Hardware Implementation

Given the proposed architecture for this IoMT, in the following sections we will discuss the details of its hardware implementation.

### 4.3. Sensing Probe

The main component for the proposed IoMT is the sensing probe, and since there is no such probe available in the market, we designed a new probe for this purpose. The anorectal probe needs to be equipped with force sensors to detect changes in force (anal sphincter) and lumen pressure (rectum). After considering several solutions—including force sensors, strain gauges, and air pressure sensors—we selected the FlexiForce A101 sensor [35]. The A101 sensor was selected based on sensor size (3.8 mm diameter) for easy integration into probe casing, and sensing range, which is within clinical requirements (41–408 g/cm^2^ and 30–300 mmHg). Controlled load testing (50–450 g) demonstrated its suitability for application as the anal sphincter sensor. However, the FlexiForce sensors alone were not capable of measuring pressure, as our pressure chamber tests did not yield any changes in sensor value. To achieve pressure measurements, the sensor was mounted on a 12 mm rod and sealed inside an airlock bag with compressed foam on top, this ensured the internal air pressure (airlock bag) remained at atmosphere pressure, such that the volume reduced when an external air pressure was applied. This setup resulted in the desired results, with good sensitivity in output measurements when 10–200 mmHg of pressure was applied; additionally, it maintained capabilities to detect contact force. This sensor layout was used for our prototype design.

**Figure 3:**
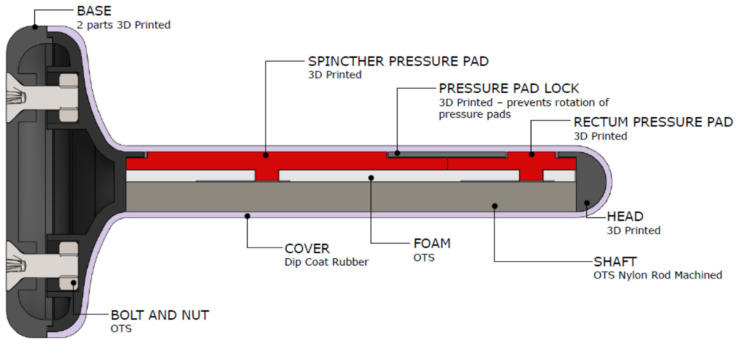
Sensing probe design and key components.

The prototype was developed with probe dimensions of 85 × 10 mm, attached to a flared base, as can be seen in Figure 3. The key design features are the following:

(1) Pressure pads: Made from 3D printable, high-resolution castable wax resin to ensure uniform measurement of force over the surface of the sensors. They captured force over a large surface area and concentrated that force to the sensor, such that we can miniaturize the sensor and obtain a higher measurement for a given applied force or pressure.
(2) Foam: Made from neoprene, to provide a spring force against the pad to ensure the pressure is released from the sensor when the contraction of the muscles and cavity is released.
(3) Pressure pad locks: These ensure uniform measurement of pressure over the surface of the pressure pad by maintaining alignment of pressure pad to the sensor.
(4) Cover and core: Dip coat rubber was used for the cover to allow for a soft surface, and the probe shaft was a nylon rod, machined to specification.

### 4.4. Networking

**Figure 4:**
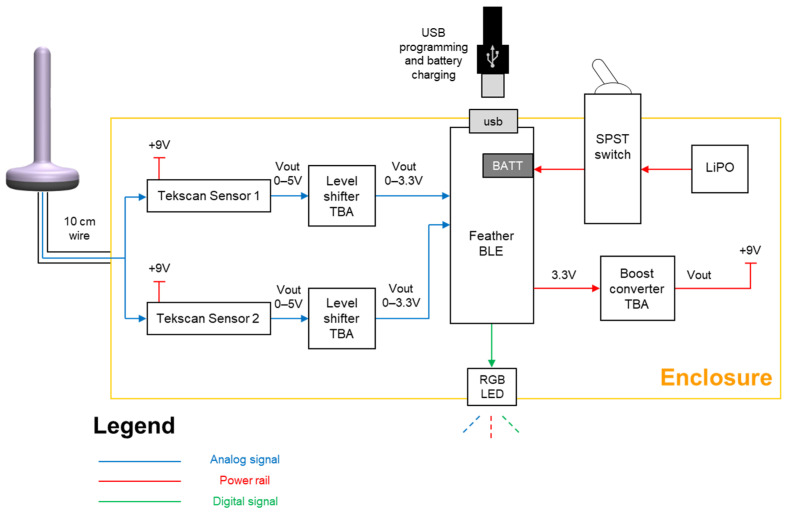
Circuit diagram of the networking and battery module.

Given the sensing probe, we needed to design a networking and battery component to power the sensors, collect user data, and send it to the mobile app. For the prototype stage, we designed a small circuit using the Adafruit Feather nRF52840 Express [36], which is a new microcontroller with Bluetooth low energy (BLE) and native USB support. It includes the ARM Cortex-M4F processor, 1 MB flash, and 256 KB SRAM. BLE has been selected as it is a short-range wireless technology with low power consumption, which is the best option for a battery-operated device. The circuit diagram and its components are illustrated in Figure 4, with indication of analogue signals, digital signals, and power rail.

As can been seen, the sensor data from the sensing probe will be sent to the microcontroller as analogue signals and will be converted to digital values. The circuit power comes from a lithium polymer (LiPO) rechargeable battery with capacity of 400 mAh with an on/off switch and a RGB LED indicator. In order to charge the battery and program the microcontroller, a micro-USB port was created. The circuit can be placed in a small enclosure and connect to the sensing probe with a short wire. Firmware was developed to relay probe sensor information to be sent via Bluetooth to the mobile app. The measurement range was calibrated against the pressure values from HD-ARM, the clinical standard for anorectal measurements. The normalized data output (unit) would be equivalent to HD-ARM pressure values 0–300 mmHg under applied pressure/force. Hence, our probe sensor data is used as the ground truth to clinically evaluate our system performance in Section 5.

### 4.5. Software Implementation

There are two software components for the proposed IoMT, including the mobile app and the cloud-based web application, which are explained in the following sections.

#### 4.5.1. Mobile App

**Figure 5:**
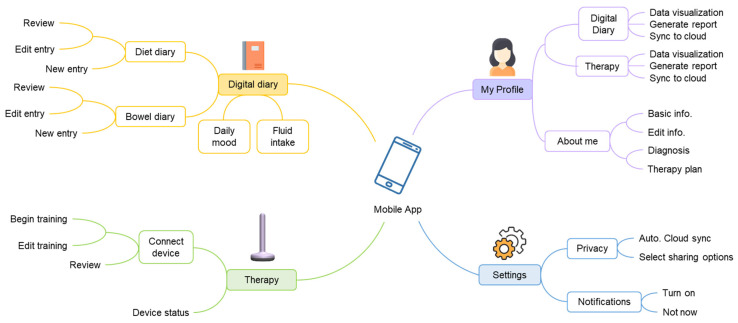
The information structure of the mobile app.

An IoMT gateway and interface for the user, a mobile app, was developed to provide users with real-time visual feedback during training and guidance on therapy activities. In addition, we have developed a digital version of the physical diary, used to record clinical indicators to evaluate therapy progress and success. The specific information structure of the mobile app is presented in Figure 5.

The user profile information includes a patient’s personal information as well as their relevant medical history. The digital diary data is collected manually on a daily basis, using the mobile app. Diet entries are added and shown as total calories and nutrient composition (fibres, protein, sugar, and fat). Bowel entries are presented as a bowel score, which is indicative of symptom severity. During each training session, quantitative BF parameters are recorded, such as resting pressure, maximum squeeze force, and % anal sphincter relaxation. Users can view these parameters to assess their progress.

**Figure 6:**
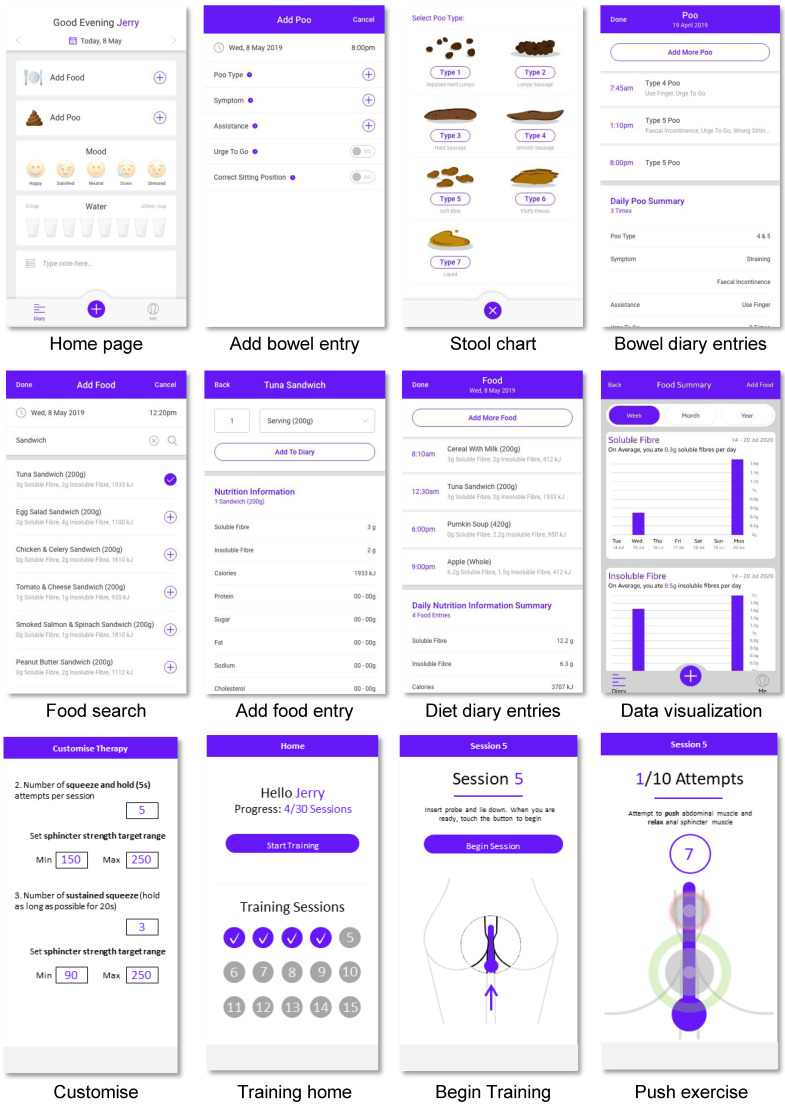
Examples of the mobile app’s user interface designs.

For the interface design, the principles of simplicity, user friendliness, and usability are adopted. As shown in Figure 6, the interface includes the home page, a bowel diary (symptoms, stool chart), a diet diary (daily caloric intake, nutrient information), a therapy record, and a BF therapy section (training regime and visual feedback). The BF training is tailored to the user’s diagnosis and set target goals, for example, to improve anal sphincter squeeze force to within normal range. A series of training sessions are scheduled over treatment period, involving coordinated anal sphincter and rectal muscle exercises. The user’s muscle tone is represented in real-time by two grey circles (anal sphincter and rectum), with stronger contractions reducing the circle size and relaxation increasing the circle size. Predetermined values (e.g., normal pressure ranges) are represented as green circles and the training involves users matching their grey circle to the green circles with respect to size, coordination, and timing (Figure 6).

#### 4.5.2. Cloud-Based Web Application

**Figure 7:**
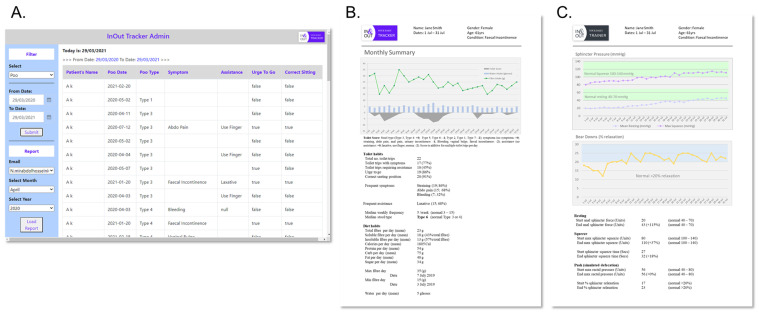
(**A**) Web application for user data search. (**B**) Example of summary report for bowel digital diary. (**C**) Example of summary report for biofeedback training.

The user input information (e.g., basic information, account authority, diary entry) are collected and stored on the app. When connected to a stable Wi-Fi connection, the user is prompted to sync their data to a cloud server for remote access by a health provider. We developed a web application using PHP and SQL server on a windows virtual machine. There is a simple web user interface, which allows health providers to search for users using their name, email, medical record number (MRN), and date of birth. Data can be filtered by type (bowel diary, diet diary, training) or date (Figure 7A). Health providers can also generate monthly summary reports from user data. The bowel diary report (Figure 7B) contains a graphical representation of relevant symptoms over the treatment period, which is seen as the primary outcome measure of BF success. The training report (Figure 7C) contains physiology measurements over each training session, these are secondary outcome measures to assess compliance and quality of exercises performed.

## 5. Performance Evaluation

In this section, we will evaluate the feasibility of the proposed IoMT system to provide information regarding the set of anorectal exercise parameters that can aid with BF training. According to the American Neurogastroenterology and Motility Society, BF exercises should include the following manoeuvres: rest, squeeze, squeeze and hold, and push [9]. Users are required to perform these manoeuvres, while the trainer (therapist or IoMT) identifies these manoeuvres based on key parameters and provides appropriate feedback to the user.

Our main aim is to find a simple, but sufficient IoMT setup that would provide enough information about key exercise parameters in BF therapy. A similar study [37], evaluating the accuracy and precision of sensor data for real-time therapist feedback, utilized a case study with a single optimal user (elite professional swimmer) performing various therapeutic swimming exercises.

**Figure 8:**
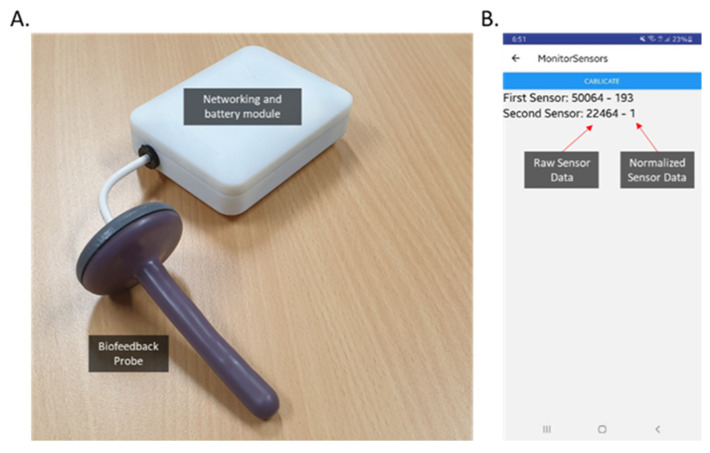
(**A**) The new IoMT sensing probe attached to networking/battery module. (**B**) The mobile app showing real-time output data from sensing probe. Raw sensor data and normalized values from first sensor (anal sphincter) and second sensor (rectal) are displayed.

We have designed a case study where a healthy individual performs BF training manoeuvres with the IoMT. Information obtained from the anorectal sphincter and rectum will be compared against conventional clinical equipment (HD-ARM) over several days of use. As mentioned earlier, a system prototype was implemented for performance evaluation, as shown in Figure 8. To our knowledge, previous home BF systems have not been directly compared with anorectal manometry in measuring BF manoeuvre parameters. To date, home BF studies have mainly used clinical indicators (e.g., bowel symptom scales and quality of life scores) [28,31,32] or pre- and post-therapy manometry studies to evaluate treatment success [18,19].

### 5.1. IoMT Clinical Feasibility Study

The new IoMT (Figure 8) was used to perform anorectal BF manoeuvres in a healthy volunteer (34-year-old male) in 3 sessions over consecutive days. The IoMT sensing probe was paired with the mobile app and calibrated (zeroed) at room temperature and atmospheric pressure. A condom sheath was placed over the probe and sufficient water-based lubricant was used for a comfortable insertion. There was approximately 10 mm of distance between the anal verge and the device base. The user lay on their side (left lateral position) and inserted the probe into the anal canal. Correct positioning is confirmed by performing a quick anal sphincter squeeze to see the corresponding increase in the anal sensor value. The user underwent a 20 s rest period before performing 3 squeeze and holds, and 3 simulated defecation manoeuvres. The resting pressure was calculated from the averaged anal sphincter values recorded in 5 s intervals over 20 s. The maximum squeeze values and endurance (time to return to baseline) were recorded. Defecation dynamic parameters recorded include the recto–anal pressure gradient (pressure difference between the rectal and anal pressures taken over 2 s at the highest recto anal pressure gradient during the push manoeuvre) and the percentage of anal relaxation (residual anal pressure/anal resting pressure) × 100, where residual anal pressure is taken over 2 s.

The volunteer repeated the anorectal BF manoeuvres with HD-ARM (ManoScan AR, Medtronic Inc., Shoreview, MN, USA) in accordance with standard protocol [24]. BF manoeuvre parameters from the HD-ARM procedure were obtained using ManoView AR v3.0 software (Medtronic). The intra-rectal pressures were recorded from a single rectal HD-ARM sensor. Anal sphincter pressures were recorded using the eSleeve option in the software, which reduces the pressures recorded across the longitudinal extent of the anal canal into a single value. At rest, during squeeze, and during rectal distention, the eSleeve identifies the highest of all the pressures recorded by the anal sensors at every point in time. This eSleeve value is used to calculate the average and maximum anal resting pressure and the maximum squeeze pressure over 20 s during these manoeuvres.

### 5.2. Results and Discussion

**Figure 9:**
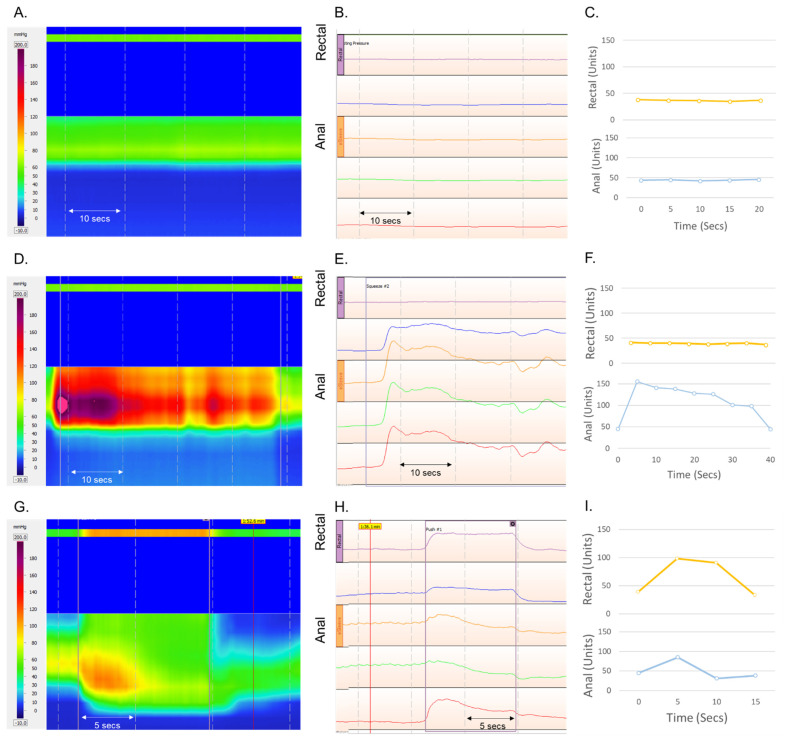
Biofeedback manoeuvres profiles depicted using high-definition anorectal manometry (HD-ARM) presented as a detailed topographical plot and waveform plot (rectal and eSleeve sensor outputs labelled), and new IoMT graphically presented as units over time in rectal and anal sensors. (**A**–**C**) Resting baseline pressures over 20 s. (**D**–**F**) Squeeze and hold manoeuvre up to 30 s. (**G**–**I**) Simulated defecation, push manoeuvre.

The anorectal BF manoeuvre values obtained from the new IoMT and HD-ARM are presented in Table 3, along with clinically defined normal values for HD-ARM [24]. The pressure profiles, relative to time and location, are presented for the new IoMT with HD-ARM as topographical plots and waveforms in Figure 9.

The presented case study showed IoMT sensors provided enough information for identification of BF manoeuvres (rest, squeeze, squeeze and hold, and push) and extraction of the most relevant parameters of BF exercises. These profiles and parameters were comparable to the clinical standard, HD-ARM waveform plots (Figure 9).

Variations in actual values between systems could be attributed to differences in probe diameter and material, given the HD-ARM probe is 17 mm wide and has a solid copper exterior, compared with the IoMT probe’s 10 mm diameter and rubber exterior. In addition, the ManoScan HD-ARM sensors are affected by differences between the environmental temperature (when calibration is performed) and the body’s temperature [37]. Although a “thermal compensation” algorithm, embedded within the acquisition software, is applied to correct for this phenomenon, recent observation with oesophageal ManoScan catheters suggest this pressure drift is related to “average pressure exposure” of a sensor during the study and may not be adequately corrected with the thermal compensation algorithm [38]. This “thermos drift” may be the cause of higher rest and squeeze manoeuvre pressures measured with HD-ARM than when measured with new IoMT. Similar, observations of higher HD-ARM parameter values have been noted when comparing with other non-high resolution manometry systems [39,40,41,42]. Further updates will use this data to improve calibration of our proposed IoMT system against HD-ARM.

With the IoMT assembly, it is important to continuously monitor and be aware of probe movement, especially after the user performs push manoeuvre, and to adjust the pressure pad to endure correct position over the anal sphincter. By contrast, in HD-ARM, this is less of an issue as the larger sensing area was less effected by movements in the probe. In addition, the new IoMT sensing area is limited to half the circumference of the probe (position of the pressure pad), whereas both HR-ARM and HD-ARM measured pressures around the circumference of the probe. This can be an issue in patients with asymmetrical anal sphincter contractility and would require positing of the new IoMT probe to capture maximal anal sphincter pressure. Overall, there was no device-related adverse event or discomfort with either system. The user experience of the proposed IoMT was subjectively more positive than HD-ARM, mainly due shorter setup time, smaller probe dimensions, soft exterior material, and freedom of movement with, no physical wires connecting to the display.

**Figure 10:**
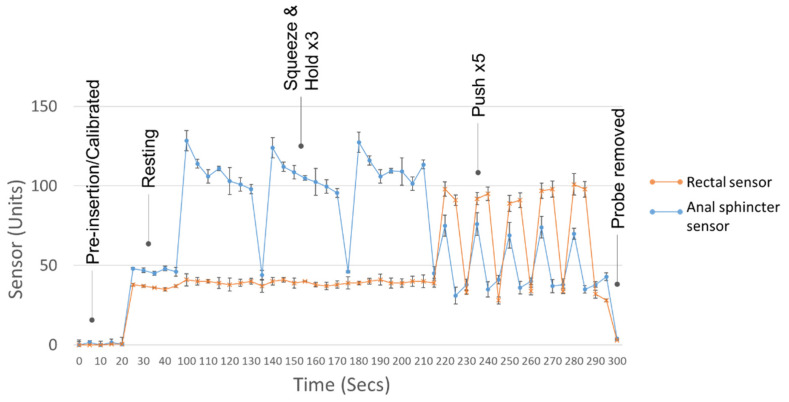
Biofeedback training session (biofeedback manoeuvres: rest, squeeze and hold, and push) performed three times over three consecutive days. Error bars are the standard deviation over the three days.

Repeat use of the proposed IoMT over three consecutive days showed similar biofeedback exercise profiles and parameters during each session (Figure 10).

There was good reproducibility between sets over consecutive days with an average SD of ±3.7 units. The technical variability is less than that of physiological changes as a result of BF therapy at 6.8–8.2 mmHg [19]. The largest variability observed was within maximum squeeze values at ±8.5 units. This observation is likely physiological, rather than technical, as prolonged squeeze manoeuvres can fatigue the anal sphincter and result in slightly lower contractile vigour. In the case of clinical BF, a 1 min recovery period is provided after each squeeze attempt to mitigate this phenomenon.

A limitation of the functionality study is that the participant did not have an underlying anorectal disorder, hence we were not able to assess the sensitivity of the new IoMT in detecting physiological changes over the course of treatment (such as improved muscle tone or coordination). However, this study gave us the confidence to plan for clinical trials to evaluate our proposed IoMT in the target population. The pilot trial will involve 20 patients with defecation disorders undergoing therapy using the proposed IoMT in a supervised clinical setting with minimal assistance from the therapist. This will assess the efficacy of treatment with the proposed IoMT, within a controlled environment. Successful outcomes will lead to a clinical trial involving 90 participants in 3 randomly assigned trial arms, as follows: (1) home-based therapy with new IoMT; (2) home-based therapy without new IoMT (education alone); and (3) clinic-based therapy with conventional equipment. This trial will evaluate the usability of the new IoMT in an unsupervised home setting and compare efficacy against current modes of treatment. Future studies will recruit patients with anorectal disorders to establish target goals and adjust the system’s sensitivity to detect changes in key anorectal physiological values.

Overall, we have demonstrated the feasibility of our proposed IoMT to quantify anorectal activities during BF therapy and generate distinct pressure profiles and values comparable to HD-ARM. We have also demonstrated reliable and consistent tracking of BF parameters over several days for the participant. Hence, we have gained confidence in the proposed IoMT for anorectal BF therapy.

### 5.3. User Evaluation of the Mobile App and Web Application

A set of usability tests was conducted among 5 users (25–52 years old) and 3 providers (specialist nurse and gastroenterologist). Users were invited to use the app for 14 days and were supplied with the physical diary currently used for clinical BF. During this period, they were tasked to record entries of their daily diet, bowel movement, symptoms, mood, and liquid intake into both the app and physical diary. Meanwhile, the providers were required to complete a set of tasks using the web application ((1) search for specific user; (2) filter for bowel diary data in specific data range; (3) generate monthly summary report). They were also provided the physical diary as a comparator. Interviews were conducted following the usability tests to discuss their user experience and identify opportunities for improvement.

All the users preferred the convenience of a digital diary over physical note taking. Though there was no difference in the average time it took for them to record an entry (24 s for both methods). The users commented on the additional nutrient information within the app in helping them monitor and manage their daily fibre intake. Minor issues were identified (random logouts, food items not in database, serving size options) and amended in the next version of the app. All providers were able to navigate and complete the tasks within a few minutes. Providers found the summary report to be a superior method of reviewing data compared with physical notes. On average, the digital diary review sessions took 2.1 min while physical notes required 5.7 min to complete (*p* value < 0.01). The summary report was also more useful in the clinical setting compared with the web interface, where the granulated data was better suited to research. The next version will streamline user search and report generation, so that providers are able to obtain summary reports in fewer steps.

### 5.4. Security and Privacy Considerations

As mentioned in Section 4.1, our proposed IoMT is divided into four different layers: sensing layer, network layer, service layer, and application layer. In each layer, there are a number of security threats, including the following: in the sensing layer, attackers can obtain physical access to an individual’s IoMT device, can read the corresponding internal memory/firmware, and can modify the configuration settings in order to fully/partially control the device. The networking layer could be potentially the major layer for security threats, as attackers can gain access to valuable information, undetected for a long duration [43]. For instance, IoMT devices, connected locally through Bluetooth, can be vulnerable to direct connection attacks. This is where the attackers use service discovery protocols, such as universal plug and play protocols, or the built-in capabilities of Bluetooth to discover IoMT devices. To overcome this type of attack, unauthenticated requests should be ignored and blocked by IoMT devices, using robust cryptographic algorithms and key management systems. To secure the Wi-Fi network, it is advised to use trusted routing mechanisms, message integrity verification techniques, and point to point encryption techniques, based on cryptographic algorithms. In the service layer, while the cloud infrastructures are highly secure, behaviour which does not follow a well-designed secure architecture could be a potential threat. Moreover, data privacy for medical information could be an issue. Edge computing [44] could address this challenge by keeping the private information close to the end user (in this case in their smart phones), and only send some data analytic requests to the cloud for long-term storage and reporting. In the application layer, there might be a number of security threats including data thefts. Using data encryption and multi-factor authentication could be solution to mitigate these threats. Interested readers can refer to [45,46] for more comprehensive surveys about security and privacy issues and solutions in IoMT systems.

## 6. Conclusions

This paper presents the development of a new IoMT for home-based BF therapy. We design, develop, and implement a new sensor configuration to measure anorectal muscle activities and provide real-time feedback to the user. We developed an IoMT gateway to allow treatment data to be accessed remotely by clinical providers, which overcomes the disadvantages of previous home BF systems. We evaluated our system against HD-ARM, and were able to achieve comparable pressure values and profiles for a series of BF training exercises. In addition, we demonstrated reliable data recording and remote access through cloud servers. In summary, we determined the feasibility of the new IoMT in quantifying anorectal muscle actives and demonstrated its capability to monitor progress remotely. As future work, we are planning to conduct a comprehensive clinical trial of individuals with functional anorectal disorders and evaluate the efficacy of the new IoMT in delivering anorectal BF therapy. Moreover, we intend to develop a predictive model using the collected dataset to help health professionals better analyse the behaviour of patients and provide personalized feedback.

## Figures and Tables

**Table 1:** Home anorectal biofeedback system and proposed IoMT.

Device	Sensor Type	Feedback	Data Storage/Sharing	Refs
MyoTron 120 (Enting Instruments & Systems, Dorst, The Netherlands)	EMG electrode	Audio feedback	No	[31]
Home Biofeedback device (DMI Medical Limited, Shrewsbury, UK)		Light strip illumination	No	[32]
InTone MV (InControl Medical, Brookfield, WI, USA)		Numeric and light strip display	Internal data storage, downloadable to PC	[28]
Biosearch Biofeedback monitor (Biosearch Medical Product Inc., Somerville, NJ, USA)	Single (air-filled) pressure sensor	Light strip illumination	Analogue strip chart recorder	[33]
Anatoner (Protech, Bengaluru, India)	Two (air-filled) pressure sensors	Light strip illumination	No	[18]
Portable Biofeedback Device (Research Prototype, Porto Alegre, Brazil)	Single pressure transducer	Waveform on LCD display	Internal data storage, downloadable to PC	[19]
Proposed IoMT	Two force sensors	Mobile app display	In app storage, cloud platform data sharing	

**Table 2:** Anorectal biofeedback needs of the patients and clinical staff.

	Therapy	Pain Points	Needs	Service Opportunity
Patients (user)	Clinic	Treatment is time consuming; requiring multiple clinic visits to selected clinics	Improve accessibility	Home therapy
	Clinic	Therapy is embarrassing and invasive	Private and comfortable settings	Home therapy
	Home	Difficult to setup	Quick setup	Intuitive user experience (UX)
	Home	No sure if performing correct exercises	Clear training instructions	Simple training user interface (UI)
Clinical Staff (provider)	Clinic	Resource intensive; limited by number of patients they can treat at once	Time and cost saving	Home therapy
	Clinical	Poor compliance; <30% complete full therapy	Improve accessibility	Home therapy
	Home	Cannot ensure patients are performing training correctly	Remote monitoring	Data sync with cloud server
	Home	No standardization between home devices and clinical equipment	Comparable to HD-ARM	Calibrate against HD-ARM

**Table 3:** Comparison of anorectal biofeedback parameters with proposed IoMT and high-definition anorectal manometry from a healthy volunteer.

Biofeedback Manoeuvres	Proposed IoMT (Units) Mean ± SD	HD-ARM (mmHg) Mean ± SD	Normal HD-ARM Range (mmHg)
Mean resting anal pressure	48 ± 1.1	49 ± 0.8	40–70
Mean resting rectal pressure	36 ± 0.6	36 ± 0.2	30–90
Maximum anal squeeze pressure	132 ± 4.8	133 ± 4.0	100–180
Duration of sustained squeeze (s)	30 ± 0.2	30 ± 1.2	>15
Recto–anal pressure gradient	−58 ± 3.9	−59 ± 2.9	−50–−6
Anal relaxation (%)	41 ± 1.6	41 ± 1.7	20–60

## Data Availability

The data presented in this study are available on request from corresponding author.
